# Identification of a novel immune signature for optimizing prognosis and treatment prediction in colorectal cancer

**DOI:** 10.18632/aging.203771

**Published:** 2021-12-13

**Authors:** Yan Li, Yiyi Li, Zijin Xia, Dun Zhang, Xiaomei Chen, Xinyu Wang, Jing Liao, Wei Yi, Jun Chen

**Affiliations:** 1 Zhongshan School of Medicine, Sun Yat-Sen University, Guangzhou, Guangdong, China; 2 Department of Medical Oncology, Sun Yat-Sen University Cancer Center, Guangzhou, Guangdong, China; 3 State Key Laboratory of Oncology in South China, Collaborative Innovation Center for Cancer Medicine, Sun Yat-Sen University Cancer Center, Sun Yat-Sen University, Guangzhou, Guangdong, China; 4 The First School of Clinical Medicine, Southern Medical University, Guangzhou, Guangdong, China; 5 The Sixth Affiliated Hospital, Sun Yat-Sen University, Guangzhou, Guangdong, China; 6 Zhongshan Ophthalmic Center, Sun Yat-Sen University, Guangzhou, Guangdong, China; 7 Guangdong Provincial Key Laboratory of Malignant Tumor Epigenetics and Gene Regulation, Sun Yat-Sen Memorial Hospital, Sun Yat-Sen University, Guangzhou, Guangdong, China; 8 Guangdong Engineering and Technology Research Center for Disease-Model Animals, Laboratory Animal Center, Zhongshan School of Medicine, Sun Yat-Sen University, Guangzhou, Guangdong, China; 9 Key Laboratory of Tropical Disease Control of the Ministry of Education, Sun Yat-Sen University, Guangzhou, Guangdong, China; 10 Center for Precision Medicine, Sun Yat-Sen University, Guangzhou, Guangdong, China

**Keywords:** colorectal cancer, immune signature, prognosis, immunotherapy, precision medicine

## Abstract

Background: Globally, colorectal cancer (CRC) is one of the most lethal malignant diseases. However, the currently approved therapeutic options for CRC failed to acquire satisfactory treatment efficacy. Tailoring therapeutic strategies for CRC individuals can provide new insights into personalized prediction approaches and thus maximize clinical benefits.

Methods: In this study, a multi-step process was used to construct an immune-related genes (IRGs) based signature leveraging the expression profiles and clinical characteristics of CRC from the Gene Expression Omnibus (GEO) database and the Cancer Genome Atlas (TCGA) database. An integrated immunogenomic analysis was performed to determine the association between IRGs with prognostic significance and cancer genotypes in the tumor immune microenvironment (TIME). Moreover, we performed a comprehensive *in silico* therapeutics screening to identify agents with subclass-specific efficacy.

Results: The established signature was shown to be a promising biomarker for evaluating clinical outcomes in CRC. The immune risk score as calculated by this classifier was significantly correlated with over-riding malignant phenotypes and immunophenotypes. Further analyses demonstrated that CRCs with low immune risk scores achieved better therapeutic benefits from immunotherapy, while AZD4547, Cytochalasin B and S-crizotinib might have potential therapeutic implications in the immune risk score-high CRCs.

Conclusions: Overall, this IRGs-based signature not only afforded a useful tool for determining the prognosis and evaluating the TIME features of CRCs, but also shed new light on tailoring CRCs with precise treatment.

## INTRODUCTION

Colorectal cancer (CRC) is the third most frequently occurring cancer and the second leading cause of cancer-related deaths worldwide in 2018 [1]. The current therapeutic options for CRC include endoscopic and local surgical excision, downstaging preoperative radiotherapy and systemic therapy, extensive surgery, local ablative therapies for metastases, palliative chemotherapy, targeted therapy, and immunotherapy [2]. It’s a highly heterogeneous disease on account of accumulating mutations attributed to environmental and genetic factors for years, which makes prognostic prediction and treatment to be exceedingly challenging [3, 4]. Therefore, there is an urgent need to incorporate other important elements to guide personalized therapies for CRCs, thereby improving the survival and prognosis of CRCs.

In recent years, a myriad of publications have highlighted that the tumor immune microenvironment (TIME) is critically involved in cancer initiation and progression [5, 6]. For example, tumor-infiltrating lymphocytes (TILs) were in close interaction with relapse and mortality prediction in CRC [7–9]. Besides, immune checkpoint inhibitors (ICIs) targeted programmed cell death protein 1 (PD-1)/programmed Cell Death-Ligand 1 (PD-L1) have been proved effective in the treatment of CRC [10, 11], revolutionizing oncotherapy to a great extent. Michael J et al. have demonstrated that a combination of PD-1 inhibitor (nivolumab) and cytotoxic T-lymphocyte-associated protein 4 (CTLA-4) inhibitor (ipilimumab) has comparatively better efficacy and is a promising new therapeutic option for metastatic DNA mismatch repair-deficient and microsatellite instability-high (dMMR–MSI-H) CRCs [12, 13]. As the most widely investigated marker, tumor PD-L1 expression might be useful as a predictive marker of response to anti-PD-1 treatment for non-small cell lung cancer (NSCLC), gastric cancer and gastroesophageal junction tumors [14, 15]. But in CRC, PD-L1 expression wasn’t tightly associated with the response or survival in the recent studies [16]. Thus far, several other biomarkers of potential response have been demonstrated, including high tumor mutation load [17, 18], high immunoscore [19, 20], and *POLE* mutation [21, 22]. However, these biomarkers that guided the use of ICIs in patients with CRC are not always consistent in clinical practice. For example, high immunoscore were also substantiated in pMMR–MSI-L CRCs, raising queries of whether single immunophenotype might robustly predict immunotherapy benefit [23]. Consequently, integrative immunogenic features of the TIME might be more precise in predicting immunotherapeutic response than either feature alone. In conclusion, developing a novel immune signature complementary for the currently established signatures is of great importance to optimize individual specialized immunotherapy for CRC patients.

Within the past decade, studies have aimed at elucidating the roles of immune-related genes (IRGs) in CRC. Li et al. have constructed an IRGs signature leveraging expression profiles and clinical characteristics from the GEO database and the TCGA database. Robust prognostic ability was demonstrated, meanwhile, the enrichment with cytotoxic immune cells as well as depletion of myeloid-derived suppressor cells (MDSC) and regulatory T cells (Tregs) were estimated in low-risk signature CRCs [24]. Lin et al. also comprehensively analyzed the role of IRGs in CRCs via the TCGA dataset, reporting a higher prognostic performance of 10 IRGs based signature in CRC and the infiltration degree of various immune cells [25]. Nevertheless, there has been no IRGs signature that comprehensively evaluates the TIME and predicts prognostic significance in conjunction with the response to chemotherapeutic and immunotherapeutic options of CRC.

In this study, we aimed at establishing a novel IRGs-based signature for CRC to investigate the interplay between colorectal immune activity profile and oncology genotype. Through systematic *in silico* analysis based on the constructed signature, we discovered that the IRGs risk score for CRC was associated with overall survival (OS), clinicopathological factors, and immunophenotypic characteristics. Moreover, we also assessed the efficiency of this IRGs signature in identifying chemotherapeutic compounds and immunotherapy with subtype-specific efficacy.

## MATERIALS AND METHODS

### Data preparation

Processed RNA-Seq FPKM data and clinical information of CRC were collected from the TCGA database. The TCGA colon adenocarcinoma (COAD, n = 512) cohort and rectum adenocarcinoma (READ, n = 177) cohort were obtained from the GDC data portal (https://portal.gdc.cancer.gov/repository). For validation, the expression profiles and detailed clinical information of GSE39582 (including 562 CRC samples based on GPL570 platform) were retrieved from the GEO database (https://www.ncbi.nlm.nih.gov/geo/). The immune gene lists were obtained from the ImmPort database (https://immport.niaid.nih.gov) [26] and overlapping genes from the TCGA dataset were defined as IRGs in the current study and extracted for the subsequent analysis.

To analyze the drug sensitivity in human CRCs, GSE17538 (including 232 CRC samples based on the GPL570 platform) was obtained from the GEO database. The expression profiles of human cancer cell lines (CCLs) were achieved from the Broad Institute Cancer Cell Line Encyclopedia (CCLE) project (https://portals.broadinstitute.org/ccle/) [27]. Drug sensitivity data of CCLs were extracted from the Cancer Therapeutics Response Portal (CTRP, https://portals.broadinstitute.org/ctrp) and PRISM Repurposing dataset (https://depmap.org/portal/prism/). The PRISM is composed of sensitivity data for 1448 compounds over 482 CCLs and the CTRP comprises of sensitivity data for 481 compounds over 835 CCLs. The area under the dose-response curve (area under the curve-AUC) values as a measure of drug sensitivity are presented in both two datasets, with lower AUC values indicating higher drug sensitivity. After the exclusion of compounds with more than 20% of missing data, the missing AUC values were imputed by K-nearest neighbor (k-NN) imputation.

To investigate the response to immunotherapy, tumor expression profiles of six immunotherapeutic cohorts were obtained. Roh et al. (2017) dataset contained melanoma patients receiving CTLA-4 or PD-1 blockade therapy was extracted from the supplementary files of reference [28]. Gene expression profiles and survival information of metastatic melanoma patients treated with CTLA-4 immuno-inhibitor were obtained from the work of Van Allen et al. (2015) [29]. The data of Ulloa Montoya et al. (2013) cohort with non–small-cell lung cancer (NSCLC) patients who were administered MAGE-A3 antigen-specific immunotherapy were downloaded from (GSE35640) [30]. The dataset of Hugo et al. (2016) included metastatic melanoma patients treated with anti-PD-1 agents was acquired from GSE78220 [31]. Moreover, patients with metastatic urothelial cancer treated with PD-L1 blockade therapy from the IMvigor210 cohort [32] and the dataset of Snyder et al. (2017) [33] were also enrolled.

### Construction of the IRGs signature for CRC

Differentially expression genes (DEGs) between tumor and normal samples from CRC patients from TCGA-COAD and TCGA-READ cohorts were first screened by limma package [34] with a cutoff value of false discovery rate (FDR)-adjusted P-value < 0.01 and log2 | fold change (FC) | > 1. Then differentially expressed IRGs between the aforementioned CRC tumor and normal tissues were obtained using a strict criterion of FDR-adjusted P-value < 0.01 and log2 | fold change (FC) | > 2. Heatmaps were plotted utilizing pheatmap package and volcano plots were generated via R software.

The CRC tumor samples from the TCGA cohort were enrolled as the training cohort to construct the IRGs signature. Univariate Cox regression analysis of differentially expressed IRGs was performed by survival package in R. The prognosis-related IRGs (PRIRGs) were selected by a cutoff value of P < 0.01. To avoid the overfitting of IRGs signature and to delete highly correlated genes, dimensionality reduction analysis was conducted by the Least Absolute Shrinkage and Selection Operator (lasso) regression through survival and glmnet R packages using gene expression profiles and overall survival data. Lambda.min was set up as cutoff point to produce minimum mean cross-validated error and genes with the highest lambda values were selected for further analysis. Subsequently, multivariate Cox regression was harnessed to develop an IRGs signature based on the expression of these genes and to calculate the risk score for signature: $\Sigma_{i=1}^{n}\;\beta i*xi$ (β represents the regression coefficient, and *x* stands for gene expression value). The training cohort samples were stratified into high- and low- risk groups according to the median value of the IRGs signature risk score.

Survival analysis for high- and low-risk subgroups was then carried out using Kaplan-Meier methods and the log-rank test was used to determine the statistical significance of differences. Time-dependent receiver operating characteristic (ROC) curves were also generated leveraging survivalROC R package to validate the prognostic ability of the IRGs signature. The IRGs signature obtained from the training cohort were used to assign the validation cohort as well as datasets containing therapeutic information into high- and low- risk score subtypes. Furthermore, to assess the independence of the constructed signature’s predictive ability, we performed univariate analysis on the IRGs signature using all clinical factors in the training and validation cohort. The hazard ratio (HR) was measured by a Cox regression model using survival package in R and forest plots were drawn.

### Gene set enrichment analysis

Gene set enrichment analysis (GSEA) in the CRC cohorts was carried out by clusterProfiler R package [35]. Fold change (FC) of each gene between subgroups was firstly produced by limma R package, and input genes were then ranked in descending order according to the logFC values. GSEA was subsequently applied to enrich 50 hallmark gene sets (h.all.v7.0.symbols) achieved from the Molecular Signatures Database (MSigDB) [36]. Enrichment significance was evaluated using default settings and FDR adjusted P-value < 0.05 was considered significantly enriched. The single sample gene set enrichment analysis (ssGSEA) [37] implemented in R package GSVA, was adopted to calculate the normalized enrichment score (NES) of immune-related signatures in the training and validation cohorts.

### Collection of cancer- and immune-related data

Four consensus molecular subtypes (CMS) CMS1-CMS4 of training and validation group were classified through CMScaller R package [38]. Six immune subtypes C1-C6 of CRC were sorted out by ImmuneSubtypeClassifier package in R [39]. The ESTIMATE score, immune score, stromal score, and tumor purity for each CRCs were quantified by the estimate algorithm [40]. The cytolytic activity (CYT) score was yielded as the geometrical mean of the GZMA and PRF1 for evaluating the cytolytic T-cell activity in TIME [17], 78 immunomodulators [39], 8 fibroblasts [41], and 335 gene signatures of 10 oncogenic pathways [42] were extracted from the previously published literature, respectively.

CIBERSORT package in R was employed to estimate the proportion of 22 immune cell types based on expression profiles [43], with the perm set at 1000. The infiltration levels of 24 immune cell types in the CRC TIME were further calculated by ssGSEA implemented with deconvolution approach, applying gene signatures expressed by specific immune cell populations [44].

### Estimation of drug response in clinical samples

Large-scale drug screening and molecular data across hundreds of cancer cell lines in pharmacogenomic databases of CTRP and PRISM make it possible for precise drug response prediction in clinical samples. Ridge regression model that located in the R package pRRophetic [45] was used to evaluate the drug responses in clinical samples, with a robust predictive power assessed by 10-fold cross-validation in default. The prediction model was merely employed on expression profiles and drug response data of solid CCLs, and the AUC value of each agent in each clinical sample was ultimately estimated. Agents with NAs in more than 20% of the samples and hematopoietic as well as lymphoid tissue-derived CCLs were excluded. Subclass mapping (SubMap) analysis (Gene Pattern modules, https://cloud.genepattern.org/), which can assess the similarity of molecular subtypes between independent patient cohorts based on mRNA expression matrix, was utilized to determine the potential immunotherapeutic benefit of distinct subtypes employing the available clinical response data and gene expression profiles from six immunotherapy datasets.

### Statistical analysis

R statistical software (version 4.0.2) was implemented for all statistical analyses. The evaluation of normality distribution within continuous variables was performed by Shapiro-Wilk test. Comparison of a continuous variable in two or more than two groups was conducted by parametric test (Student’s t-test or analysis of variance, respectively) if the variable was normally distributed, otherwise, nonparametric test (Wilcoxon rank-sum test or Kruskal-Wallis test) was performed. Correlation between two continuous variables was evaluated by either Pearson’s r correlation or Spearman’s rank-order correlation. For all statistical analyses, unless otherwise noted, a two-tailed P-value < 0.05 was defined as statistically significant.

## RESULTS

### Construction of IRGs signature in CRC cohorts

A total of 638 CRC and 51 adjacent normal tissues were acquired from the TCGA database. To establish a predictive IRGs signature, we performed differential expression analysis of genes and IRGs between tumor and normal tissues. A total of 3741 DEGs were identified, including 2,502 upregulated genes and 1,239 downregulated genes (Supplementary Figure 1A, 1B). 2,483 IRGs were also collected from the ImmPort database (Supplementary Table 1). Fulfilling the screening criteria, 294 differentially expressed IRGs were obtained, containing 99 upregulated IRGs and 195 downregulated IRGs (Supplementary Figure 1C, 1D). In total, 606 CRC samples with complete gene expression profiles and intact follow-up information from the TCGA database were enrolled for establishing IRGs signature in the training cohort.

To determine the IRGs related to tumorigenesis and development in CRC, univariate Cox regression analysis was implemented on the differentially expressed IRGs in the training cohort (P < 0.01), and 11 PRIRGs in all were obtained (Supplementary Figure 1E). Moreover, lasso regression was conducted to lessen the number of PRIRGs, and eight PRIRGs were thus filtered out (Supplementary Figure 1F, 1G and Supplementary Table 2). Through multivariate Cox regression analysis, seven-IRGs based signature was ultimately established, as depicted in Supplementary Table 3. The formula for calculating risk score is: Risk score = 0.139 х Exp_FABP4_ + 0.176 х Exp_AMH_ + 0.207 х Exp_GRP_ + 0.211 х Exp_INHBB_ - 0.691 х Exp_NRG1_ + 0.274 х Exp_UCN_ + 0.366 х Exp_MC1R_. Among these IRGs, NRG1 exhibited a negative coefficient, implying that it could be considered as a protective factor for CRCs; on the contrary, FABP4, AMH, GRP, INHBB, UCN, and MC1R possess positive coefficients, implying poor prognoses in CRCs with overexpression of these six genes.

**Figure 1:**
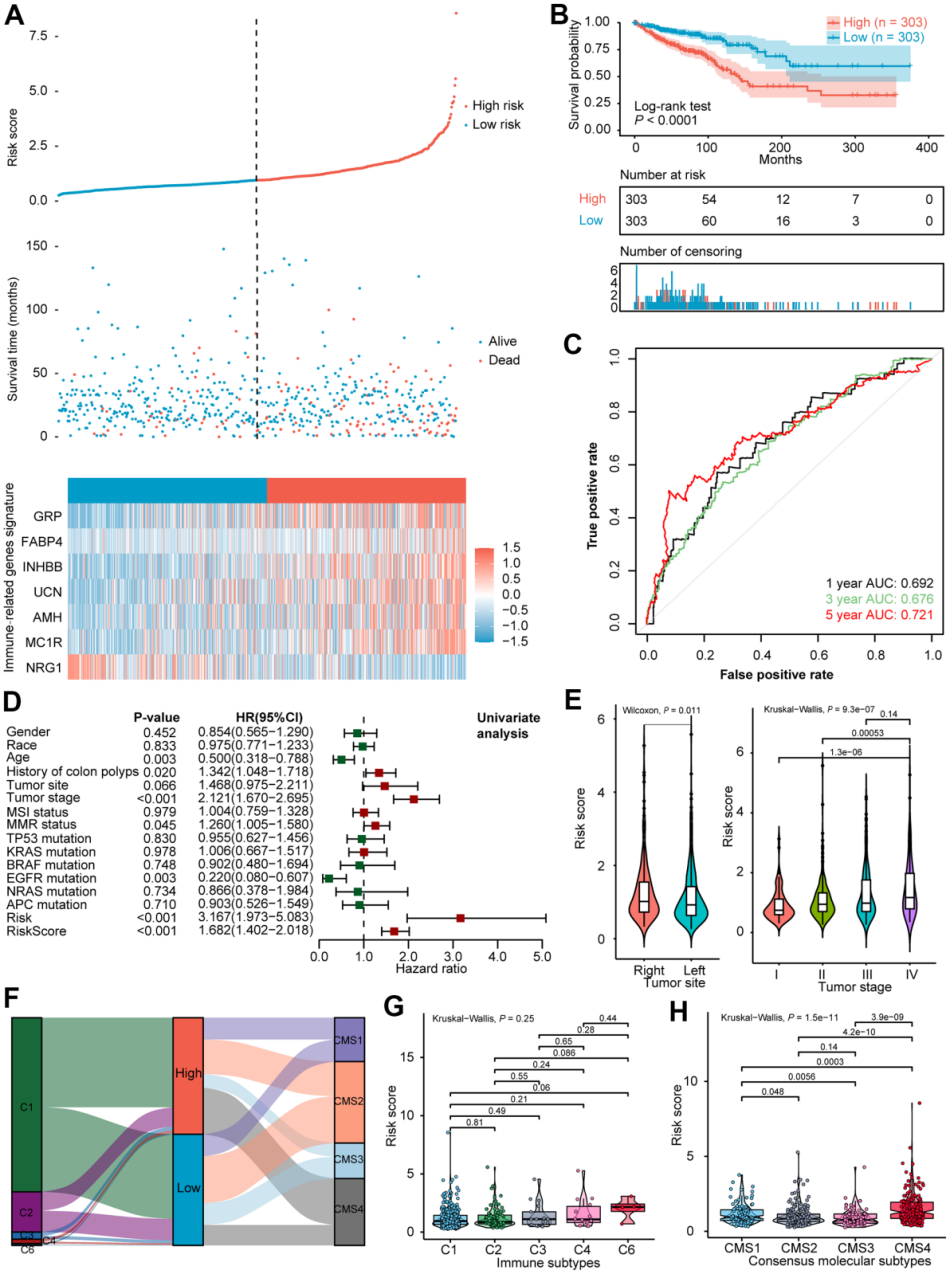
**Exploration of the predictive power and clinical characteristics of seven IRGs signature in the training cohort.** (**A**) Distribution of risk score, survival status, and expression of seven IRGs of CRCs. (**B**) Kaplan-Meier survival curve of the high- and low- risk subgroups. (**C**) ROC curve analysis of IRGs. (**D**) Univariate Cox analysis of prognostic factors and OS of CRCs. (**E**) Violin plot illustrated the correlation between risk score and tumor site as well as tumor stage. (**F**) Alluvial diagram for the two subtypes versus different immune subtypes and CMS. (**G**) Violin plot illustrated the correlation between risk score and immune subtypes, and (**H**) CMS. AUC, area under the curve; OS, overall survival; CRC, colorectal cancer; IRGs, immune-related genes; ROC, receiver operating characteristic; CMS, consensus molecular subtypes.

According to the median value of the risk score (0.948), the 606 CRCs in the training cohort were divided into a high-risk group (n = 303) and a low-risk group (n = 303). The distribution of risk scores, survival status as well as the expression level of seven IRGs for the two subgroups in the training cohort were correspondingly displayed in Figure 1A. Kaplan-Meier survival analysis (Figure 1B) indicated dismal prognosis for patients in the high-risk group (P < 0.0001). To assess the predictive efficiency of the constructed seven IRGs signature, time-dependent ROC curves were plotted. As shown in Figure 1C, the AUCs for 1-, 3-, 5- year survival prediction was 0.692, 0.676, and 0.721, respectively.

To evaluate the performance of the seven IRGs signature, the GSE39582 dataset (n=562) was used for validation. On the basis of signature information acquired from the training cohort, CRCs in the validation cohort were also classified into high-risk group (n = 281) and low-risk group (n = 281). The distribution of risk scores, survival status, and the expression level of seven IRGs for different subclasses in the validation cohort are correspondingly displayed in Supplementary Figure 2A. Kaplan-Meier survival analysis in Supplementary Figure 2B also revealed poor prognosis in patients of high-risk score (P < 0.0001). Similarly, time-dependent ROC curves were plotted. As exhibited in Supplementary Figure 2C, the AUC for 1-, 3-, 5- year survival prediction was 0.615, 0.616, and 0.662, respectively.

### Evaluation of the IRGs signature in CRC cohorts

A detailed summary of the training and the validation cohorts selected for univariate analysis in this study is presented in Supplementary Table 4. Univariate analysis of the TCGA dataset suggested that age (P = 0.003), history of colon polyps (P = 0.020), tumor stage (P < 0.001), mismatch repair system (MMR) status (P = 0.045), EGFR mutation (P = 0.003), and risk score (P < 0.001) were significantly associated with OS (Figure 1D). Meanwhile, a high-risk score was correlated with increased age, history of colon polyps, right half of CRC, dMMR as well as EGFR mutation (Supplementary Table 4). Analogous analyses in the validation dataset showed that tumor stage (P < 0.001), KRAS mutation (P = 0.048), and risk score (P < 0.001) were closely connected with patient survival (Supplementary Figure 2D). As shown in Figure 1E, the risk score was significantly higher in right-side colorectal cancer than the left-side, and the risk score was significantly elevated as colorectal cancer progressed to an advanced stage.

**Figure 2:**
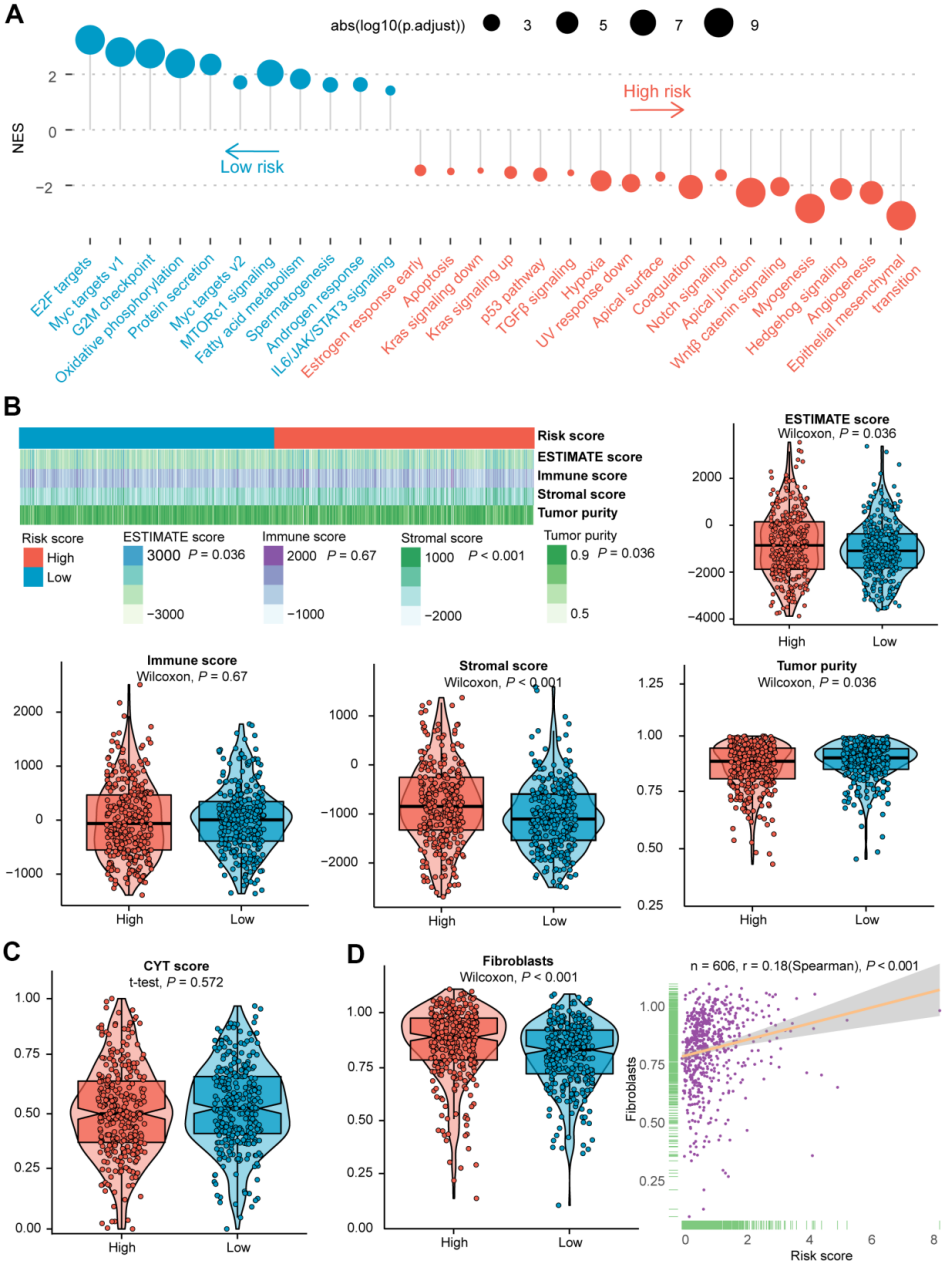
**Evaluation of the role of IRGs-based risk score in the training cohort.** (**A**) Results of GSEA of the high-risk group (red) compared with the low-risk group (blue). Color toward gray represents no statistical significance. (**B**) Heatmap and violin plots of the ESTIMATE score, immune score, stromal score, tumor purity between high- and low- risk subtypes. (**C**) Violin plot of the CYT score between high- and low- risk subtypes. (**D**) Violin plot of fibroblasts between two subtypes, and the association between risk score and the NES of fibroblasts. Statistical significance at the level of ns ≥ 0.05, * < 0.05, ** < 0.01 and *** < 0.001. GSEA, gene set enrichment analysis; CYT, cytolytic activity; NES, normalized enrichment score.

The differences in the distribution of molecular subtypes within the IRGs risk score model were also investigated. In the TCGA cohort, there was no significant difference between the risk score and the immune subtypes (Figure 1F, 1G). Similar results were manifested in the validation dataset (Supplementary Figure 2E, 2F), probably because six immune subtypes were generated by immunogenomics analyses encompassing multiple cancer types [39]. With regards to CMS, the CMS4 subtype had significantly higher IRGs risk score than the other three molecular subtypes, whereas the CMS2 subtype held the lowest risk score (Figure 1F, 1H). A significantly difference was demonstrated among the four CMSs (P = 1.5e−11). In the GEO validation dataset, CMS was likewise found distributed between high- and low- risk subgroups (Supplementary Figure 2E), and allied results (P < 2.2e−16) were displayed in violin plot (Supplementary Figure 2G). Remarkably, the international CRC Subtyping Consortium proposed that superior survival was demonstrated in CMS2 patients while CMS4 patients displayed worse OS [46], consistent with our finding that a larger proportion of long-term survivors were identified in low-risk CRCs than the high-risk subset. The GSEA of 50 hallmark gene sets indicated that up-regulated genes of the high-risk group were enriched in multiple carcinogenesis related pathways, such as epithelial-mesenchymal transition (EMT), angiogenesis, Hedgehog signaling, myogenesis, transforming growth factor-beta (TGFβ) signaling, as well as hypoxia pathway targeted HIF1A (Figure 2A and Supplementary Figure 3A). GSEA analyses revealed the enrichment of tumor proliferation-associated signatures, such as E2F targets, MYC targets, and G2M checkpoint in IRGs low-risk subgroup. Several evidences suggested that they might denote dual role of regulating anti-tumor immunity and tumor cell proliferation. It’s indicated that the E2F1/SP3/STAT6 axis induced by IL-4 promoted EMT in CRC cells [47]. Activation of IL-6/p-STAT3/c-MYC signaling was demonstrated to enhance colorectal tumor growth in a TLR4-dependent manner [48]. In addition, MYC/PVT1 signaling induced immune surveillance via CD8^+^ TILs and peripheral blood mononuclear cells in CRCs [49]. As for G2M checkpoint, *in-vitro* co-culture assays of T cells and HCT-116 colorectal cancer cells reflected that immune checkpoint TIGIT blockade suppressed G2M transit [50]. Overall, these tumor proliferation-related pathways might also exert tumor immunity associated effects on the TIME of CRC, and the underlying mechanism deserved future investigation. Metabolism-related processes consisting of oxidative phosphorylation and fatty acid metabolism, as well as immune-related signaling involved in IL6/JAK/STAT3, were observed in the low-risk group.

**Figure 3:**
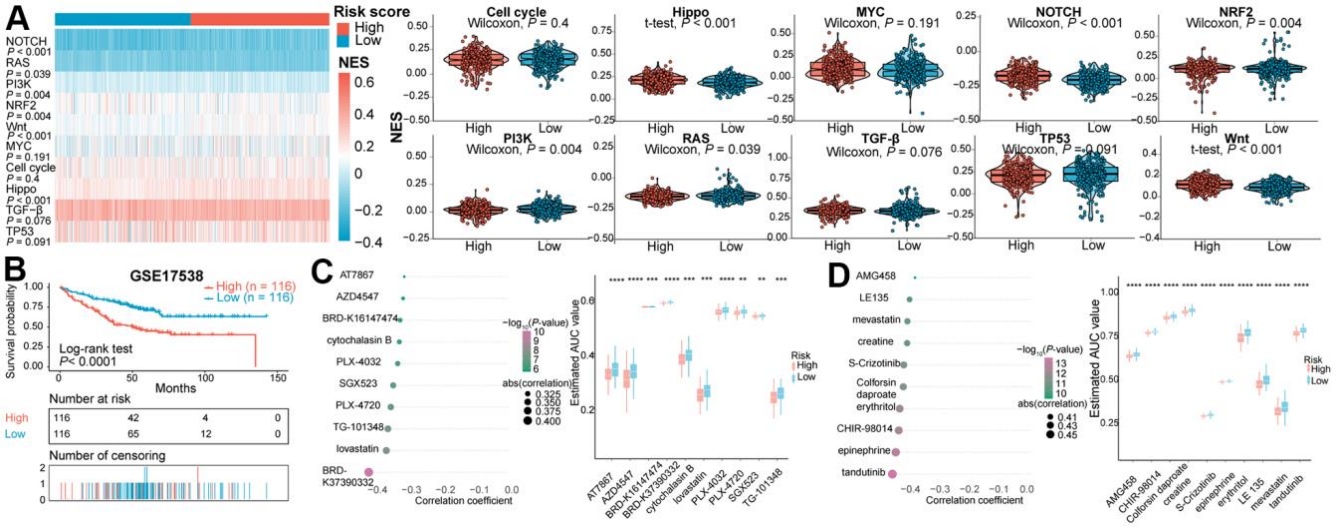
(**A**) Heatmap and violin plots of the NES of 10 oncogenic pathways between two subtypes in the TCGA cohort. (**B**) Kaplan-Meier survival curve of the high- and low- risk subgroups in GSE17538 dataset for identifying potential therapeutic agents. (**C**) Spearman’s correlation analysis and differential drug response analysis of 10 CTRP-derived compounds. (**D**) Spearman’s correlation analysis and differential drug response analysis of 10 PRISM-derived compounds. NES, normalized enrichment score.

Besides, variation in the NES values of 10 common oncogenic pathways between the two subclasses were evaluated in the TCGA COAD and READ patients (Figure 3A). The Hippo-, Notch-, NRF2- and Wnt-related pathways exhibited significantly higher NES values in high-risk subtype than in low-risk subtype. The NES values of the PI3K, RAS, and TP53-related pathways were significantly higher in the immune risk score-low subtype than in the immune risk score-high subtype. Analogous effects in Hippo-, Notch- and Wnt-associated pathways were investigated in the GSE39582 validation cohort (Supplementary Figure 5C).

### The immune landscape of the microenvironment in CRC subclasses

To further evaluate the potential molecular mechanism, the connection between four types of score produced by the ESTIMATE algorithm and risk score was also examined. Among the training dataset, a higher risk score was unveiled with elevated ESTIMATE score and stromal score, nevertheless, with decreased tumor purity (Figure 2B). Analogous patterns were found in the validation dataset except for a significantly positive correlation between risk score and immune score (Supplementary Figure 3B). Besides, no statistical significance was shown in the CYT score between the two subclasses (Figure 2C and Supplementary Figure 3C). It has been documented that fibroblasts are critical in multiple immunologic responses and inflammatory responses to tumor tissue injury [51, 52]. In the training group, the risk score was markedly correlated with the NES of fibroblasts (Spearman’s r = 0.18, P-value < 0.001, Figure 2D). Likewise, we found increased NES in high-risk subgroup of the validation cohort (Supplementary Figure 3D).

**Figure 4:**
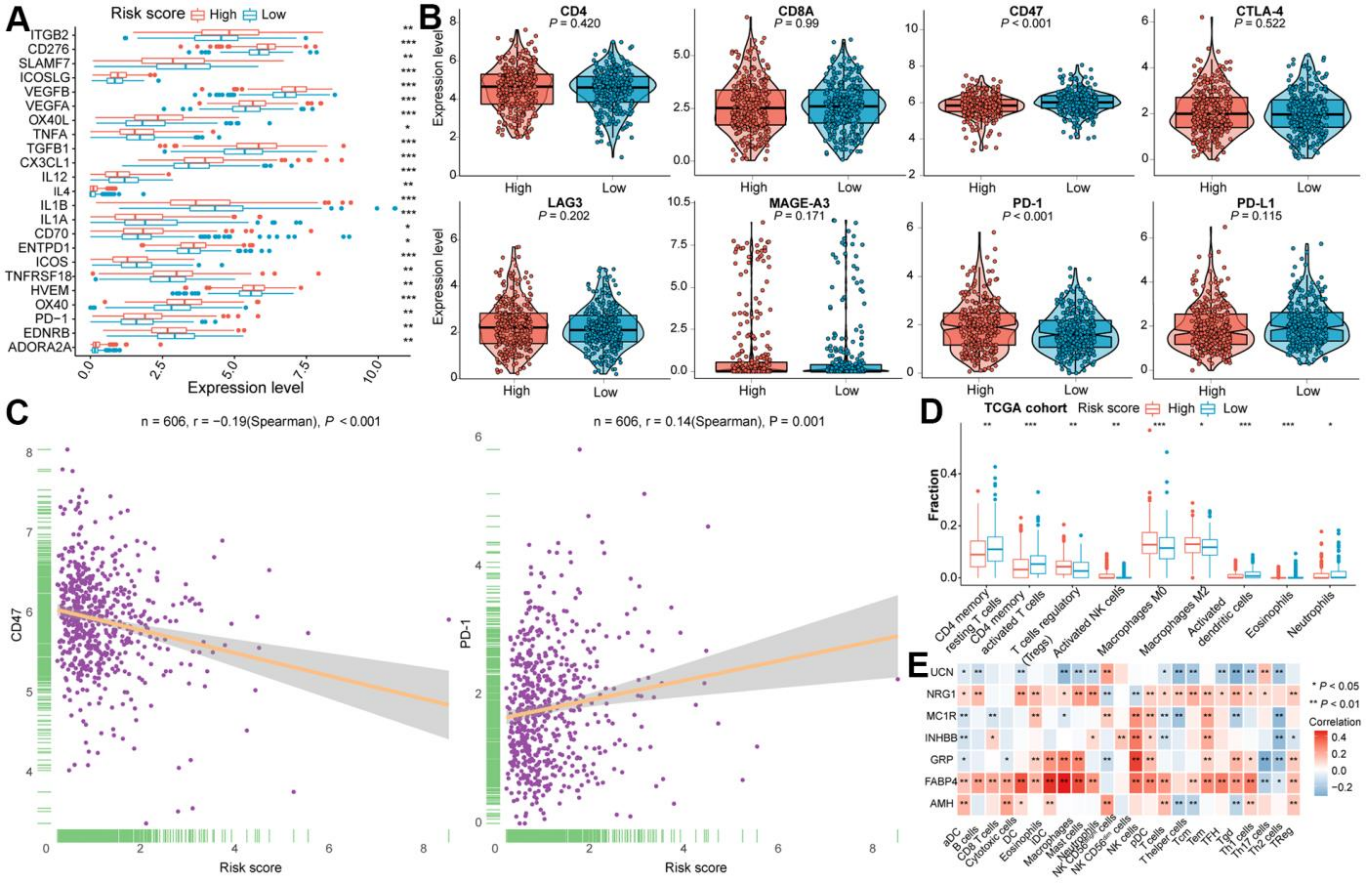
**The immune landscape of two distinctive subclasses in the training cohort.** (**A**) The differential expression level of immune checkpoint molecules between two subclasses with statistical significance. (**B**) Violin plots of the CD4, CD8A, CD47, CTLA4, LAG3, MAGE-A3, PD-1 and PD-L1 expression levels for two subtypes. (**C**) The association between risk score and CD47 as well as PD-1 expression levels. (**D**) Immune infiltration between high- and low- risk subtypes with statistical significance in the training cohort. (**E**) Correlation analysis between the expression of seven IRGs and the infiltration of immune cells. Statistical significance at the level of ns ≥ 0.05, * < 0.05, ** < 0.01 and *** < 0.001.

Immunomodulators (IM) play a determinant role in clinical oncology and plenty of IM-related agonists and antagonists are being assessed [53]. To further figure out the underlying immune modules of the constructed IRGs model, the IM gene expression level between two subgroups in two CRC cohorts was compared. Among the IMs under investigation for cancer immunotherapy, certain of them were significantly related to the risk score (Figure 4A and Supplementary Figure 4A, 4B). In addition, we deeply investigated whether the risk score was associated with the expression level of T cell markers (CD4 and CD8A) and with six vital immune checkpoint genes (CD47, CTLA-4, LAG3, MAGE-A3, PD-1, and PD-L1). As shown in Figure 4B, the expression level of PD-1 was significantly higher in CRC of the high-risk subtype, while the risk score was negatively correlated with CD47 expression. Moreover, the differences in the expression level of CD47 and PD-1 between two subtypes of the TCGA dataset were statistically significant (Figure 4C). Even though the expression level of CD4 inclined to be elevated in high-risk subclass, no statistical difference was determined in the TCGA cohort. Statistical significance was verified in the validation cohort (Supplementary Figure 4C).

To investigate whether the immunophenotype may be shaped by immune cells, the relationship of immune infiltration with subtypes in both TCGA and GEO samples was examined in depth. We found that there was conspicuous heterogeneity in immune cell population among the established classifications, consistent with previous published TILs subpopulations in CRC [54]. As illustrated in Figure 4D and Supplementary Figure 5A, the infiltrated fractions of Tregs, activated NK cells, macrophage M0, and macrophage M2 was outstandingly augmented in the high-risk group. By contraries, markedly increased infiltration of CD4^+^ memory resting T cells, CD4^+^ memory activated T cells, activated dendritic cells (DCs), and neutrophils was observed in the low-risk group. Furthermore, we evaluate the correlation between the expression of seven IRGs and the infiltration of 24 types of immune cells by ssGSEA in CRC tissues. In the TCGA cohort (Figure 4E), there was a strong correlation of the FABP4 expression with the infiltration of NK cells (Spearman’s r = 0.34, P < 0.001), GRP with NK cells (Spearman’s r = 0.41, P < 0.001) or Th17 cells (Spearman’s r = -0.32, P < 0.001), INKBB with NK cells (Spearman’s r = 0.31, P < 0.001), as well as UCN with Tgd (Spearman’s r = -0.31, P < 0.001) (Supplementary Table 5). For the training dataset and validation dataset, strong connection was confirmed between the expression of FABP4 and the infiltration of DC (Spearman’s r = 0.38, P < 0.001), iDC (Spearman’s r = 0.44, P < 0.001), macrophages (Spearman’s r = 0.46, P < 0.001), and mast cells (Spearman’s r = 0.40, P < 0.001), the expression of GRP and infiltration of macrophages (Spearman’s r = 0.30, P < 0.001) included (Figure 4E and Supplementary Figure 5B and Supplementary Table 5).

### Identification of potential therapeutic agents for CRCs with immune high-risk score

The CTRP and PRISM datasets shared 160 compounds, with 1770 compounds remained in total after removing duplication (Supplementary Table 6). Two approaches were utilized to screen candidate compounds with higher drug sensitivity in CRCs of high-risk score. By stratifying CRCs in GSE17538 dataset into high- and low- risk score subtypes based on seven IRGs (Figure 3B), the analyses were operated using CTRP and PRISM-derived drug response data, successively. First, differential drug response analysis between high- and low- risk groups was conducted to identify agents with differential estimated AUC values between subclasses (FDR < 0.05). Next, the Spearman correlation test between AUC value and risk score was adopted to identify drugs with negative correlation coefficient (Spearman’s r < −0.30 for CTRP or −0.40 for PRISM). Above analyses yielded 10 CTRP-derived agents (including AT7867, AZD4547, BRD-K37390332, Cytochalasin B, PLX-4720, SGX-523, PLX-4032, TG-101348, lovastatin, and BRD-K16147474) and 10 PRISM-derived agents (including AMG458, LE135, mevastatin, creatine, S-Crizotinib, Colforsin daproate, erythritol, CHIR-98014, epinephrine, and tandutinib). All these compounds presented lower estimated AUC values in the high-risk subgroup and a negative correlation with IRGs-based risk score (Figure 3C, 3D). Although the 20 candidate agents displayed a higher drug sensitivity in IRGs score-high patients, solely the analyses above could not draw to the conclusion that these compounds are promising treatment modality for the eradication of CRC. Therefore, an integrated literature retrieval was conducted in PubMed, DrugBank [55], and HERB [56] databases to search for the experimental and clinical evidence of candidate compounds for CRC (Supplementary Table 7). BRD-K16147474, SGX-523, BRD-K37390332, AMG458, LE135, creatine, colforsin daproate, erythritol, CHIR-98014, and epinephrine without supporting evidence for CRC were firstly excluded. Secondly, PLX-4032 [57, 58] and PLX-4720 [59] targeted B-raf^V600E^, AT7867 targeted Akt [60], tandutinib targeted Akt/mTOR pathway [61], TG-101348 targeted the JAK2/STAT3/PIM1 pathway [62], lovastatin [63] and mevastatin [64] inhibiting 3-hydroxy-3-methylglutaryl coenzyme A (HMG-CoA) reductase weren’t considered as the potential compounds for risk score-high subclass. This is because these drugs functioned inconsistently with targets enriched in the immune score-high subclass through GSEA (Figure 2A and Supplementary Figure 3A). Collectively, AZD4547, Cytochalasin B and S-crizotinib, which held true *in vitro* and *in silico* evidence, were deemed the most promising therapeutic agents for CRCs with high IRG risk scores.

### CRC subgroups have distinct responses to immunotherapy

**Figure 5:**
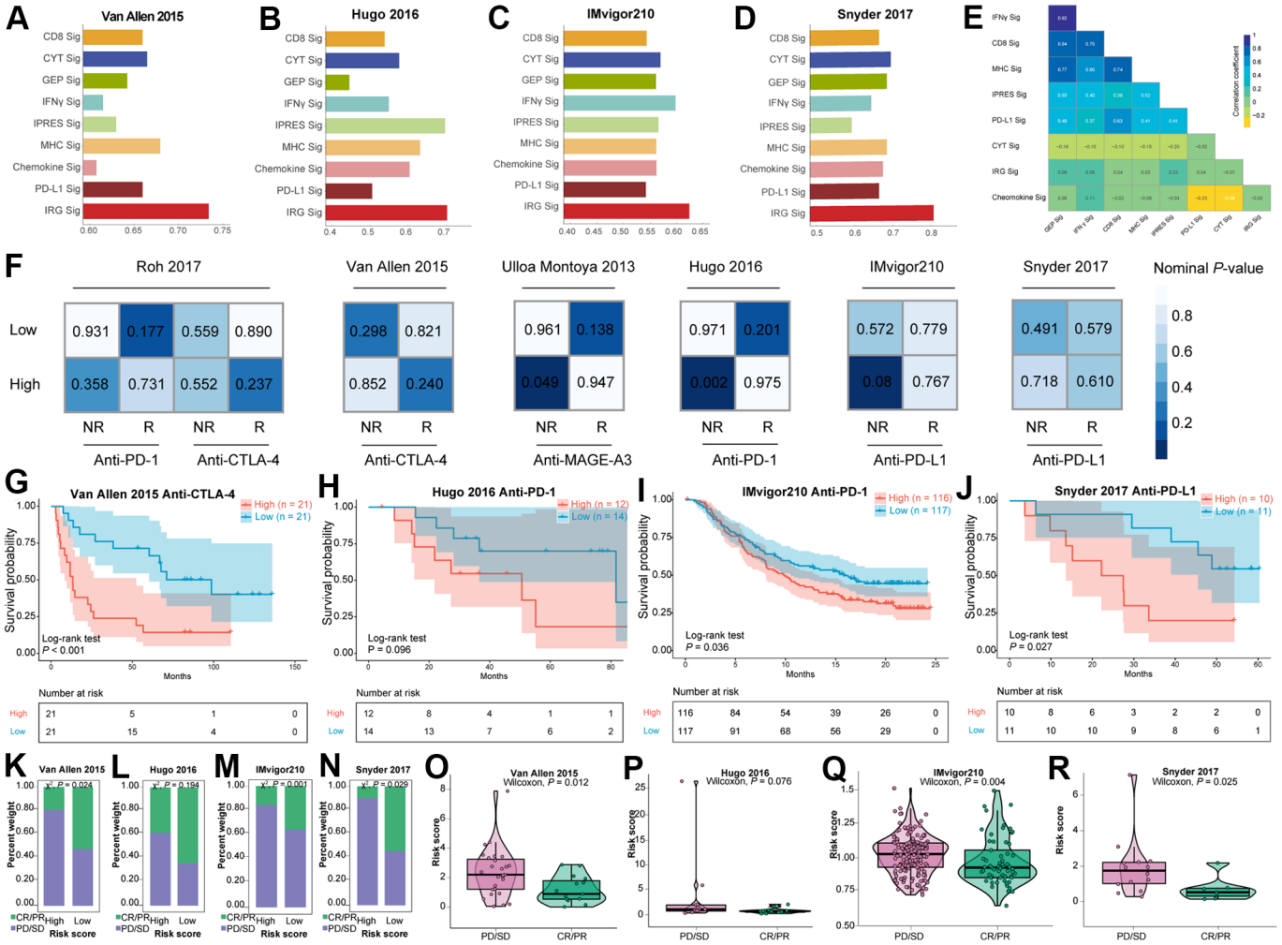
**The immunotherapeutic benefit of the IRGs-based risk score in immunotherapeutic treatment cohorts.** (**A**–**D**) Comparison of AUC values between IRGs-based signature and other eight previous published immune related signatures in four immunotherapeutic datasets. (**E**) Similarity comparison between IRGs-based signature and other seven previous signatures. (**F**) SubMap analysis utilizing six immunotherapy datasets. Kaplan-Meier survival curve of OS for patients with high- and low- risk score subtypes for (**G**) Van Allen et al. (2015) dataset, (**H**) Hugo et al. (2016) dataset, (**I**) IMvigor210 dataset, and (**J**) Snyder et al. (2017) dataset. Bar graph illustrated the treatment response to immunotherapy within high- and low- risk score subtypes in (**K**) Van Allen et al. (2015) dataset, (**L**) Hugo et al. (2016) dataset, (**M**) IMvigor210 dataset, and (**N**) Snyder et al. (2017) dataset. Violin plots illustrated the distribution of risk score for patients with different immunotherapy responses in (**O**) Van Allen et al. (2015) dataset, (**P**) Hugo et al. (2016) dataset, (**Q**) IMvigor210 dataset, and (**R**) Snyder et al. (2017) dataset.

Two different procedures were adopted in this study to identify subclass-specific candidate immunotherapies. Submap analysis was first used to find potential immunotherapeutic benefit of two subgroups through six immunotherapy datasets available with clinical response and gene expression information. As exhibited in Figure 5F, the high-risk subclass shared high similarity with anti-MAGE-A3 nonresponse group in Ulloa Montoya et al. (2013) dataset (P = 0.049) and anti-PD-1 nonresponse group in Hugo et al. (2016) dataset (P = 0.002), and the high-risk subgroup tended to be correlated with anti-PD-L1 nonresponse group in IMvigor210 cohort although no statistical significance was found (P = 0.08).

Patients received immunotherapy in Van Allen et al. (2015) dataset, Hugo et al. (2016) dataset, IMvigor210 dataset, and Snyder et al. (2017) dataset were classified into high-risk subtype and low-risk subtype using the median IRGs-based risk score as the cutoff. Then, the AUC values for classifying the responder and non-responder cases of several previous signatures, including CD8 [65], CYT [17], T cell-inflamed GEP [66], IFNγ [66], IPRES [67], MHC [68], Chemokine [69], and PD-L1 [65] signatures as well as IRGs-based signature were calculated across all the four immunotherapeutic datasets with abundant gene expression profiles. Notably, IRGs signature outperformed the other eight signatures and the AUC values exceeded 0.7 in three out of four datasets (Figure 5A–5D). The results of performance comparison in four independent datasets suggested that the predictive power of IRGs signature ranked the highest. The association across these signatures indicated that five signatures, including IFNγ, CD8, MHC, IPRES, and PD-L1 signatures correlated closely with each other (Figure 5E). By contrast, IRGs signature displayed relatively weak correlation with other signatures, implying its complementary role rather than the alternative as an immunotherapeutic indicator. Patients in the low-risk subclass presented significant longer OS than those in the high-risk subclass of Van Allen et al. (2015) dataset (log-rank test P-value < 0.001, Figure 5G), IMvigor210 dataset (log-rank test P-value = 0.036, Figure 5I) and Snyder et al. (2017) dataset (log-rank test P-value = 0.027, Figure 5J), however, no statistical difference was observed in Hugo et al. (2016) dataset (log-rank test P-value = 0.096, Figure 5H). These findings demonstrated that the lower risk score was associated with better survival outcomes in tumor patients treated with immunotherapy. Collecting immunotherapeutic response data in four cohorts mentioned above, we determined the correlation between immunotherapeutic response and risk score. It’s shown that patients in the low-risk subtype had a dramatically higher response to immunotherapy than patients in the high-risk subtype among three datasets (P-value = 0.024 for Van Allen et al. anti-CTLA-4 cohort, P-value = 0.001 for IMvigor210 anti-PD-L1 cohort, and P-value = 0.029 for Snyder et al. anti-PD-L1 cohort; X^2^ test, Figure 5K, 5M, 5N), apart from (P-value = 0.194 for Hugo et al. anti-PD-1 cohort, X^2^ test, Figure 5L). According to Van Allen et al. (2015) anti-CTLA-4 cohort (Figure 5O), IMvigor210 anti-PD-L1 cohort (Figure 5Q), and Snyder et al. (2017) anti-PD-L1 cohort (Figure 5R), violin plots revealed that the risk score was significantly decreased in patients responsive to the immunotherapeutic invention, compared to non-responsive patients. Nonetheless, no statistical significance was observed in Hugo et al. (2016) anti-PD-1 cohort (Figure 5P).

## DISCUSSION

Despite the advances in treatment, CRC is a lethal disease of great heterogeneity, prompting therapeutic optimization to prolong survival outcomes and reduce mortality. Hence, it’s essential to acquire reliable prognostic biomarkers to stratify survival risk and to predict subclass-specific therapeutic strategies. Tailoring specialized management for patients depends on personalized clinical and molecular features. Gaining insight into IRGs involved in CRC enables scientists to recapitulate the underlying mechanism of carcinogenesis in CRC and identify patients who may benefit from adaptive therapy. In this study, by exploiting a compendium of IRGs, a robust prognostic immune-based signature was built using public CRC cohorts. CRC samples with intact expression profiles and clinical characteristics were downloaded from the TCGA and the GEO database. Multivariate Cox regression was utilized to calculate the risk score for each cohort based on the seven IRGs signature independently. Besides, bioinformatic analyses were separately performed in different CRC cohorts, the normalized process was thus unneeded.

The IRGs-based risk score was found to be significantly correlated with OS in CRCs and remained significant after adjustment for clinical and pathological parameters. To characterize the TIME immune infiltration, we explored the divergent immune cell subpopulation via the CIBERSORT algorithm between subgroups. The higher pro-tumor immunocytes encompassing Tregs, macrophage M0, and macrophage M2 were observed in the high-risk group, in contrast, immune cells orchestrating anti-tumor responses including CD4^+^ memory resting T cells, CD4^+^ memory activated T cells, activated DCs, and neutrophils accumulated in the low-risk group. Imbalances in immune cell components are associated with undesirable prognosis and inferior survival outcomes in cancer patients [70, 71]. Toor et al. documented the aggregation of CD4^+^ and FoxP3^+^ TILs in CRC tissues, compared to para-carcinoma normal tissues [72]. In humans, the accumulation of Tregs within TIME is regarded as a disadvantageous prognostic factor in a plethora of cancers [73]. However, Tregs infiltration in CRC tissues is incapable of predicting the prognosis [74, 75]. Elevated infiltration of Tregs could trigger low tumor differentiation and aggrandized involvement of lymph node [74]. In contrast, enhanced Tregs densities have also been correlated with better relapse-free survival (RFS) [76, 77]. Some heterogeneous subsets of Tregs facilitate CRC progression, covering CD8^+^ Tregs [78] and RORγt^+^ Tregs [79]. Macrophage polarization plays a prominent role in tumor pathogenesis. In response to distinct microenvironments, primary macrophages (M0) migrate out of vessels and could be polarized toward pro-inflammatory (M1) macrophages or anti-inflammatory (M2) macrophages, while resting macrophages undergo diverse functional alterations [80, 81]. To some extent, M2 macrophage infiltration is closely linked with increased involvement of CRC liver metastasis and malignant lesion in the liver [82]. Moreover, cancer-associated fibroblasts (CAFs) in CRC fuel tumor-associated macrophages (TAMs) infiltration and macrophages M2 polarization in TIME, subsequently impairing the function of NK cells [83]. The increased level of CD4^+^ TILs has been deemed as favorable clinical outcome in CRC [84], highlighting the crucial role of CD4^+^ cells in regulating immune system to exert anti-neoplastic activity. In CRCs, elevated expression of Th1 transcripts is correlated with beneficial prognosis, whereas the elevated expression of Th17 transcripts is correlated with poor clinical outcome [85]. Additionally, effector and memory Th1 CD4^+^ T cells are pivotal in effective anti-tumor immunity and that CD4^+^ T cells induce more durable immune responses than CD8^+^ T cells [86]. DCs act a key role in presenting tumor antigens and eliciting tumoricidal processes of T cells [87], and activated DCs might potentiate immunotherapeutic efficacy in advanced CRCs [88]. On the contrary, inhibited functions of DCs in cancer patients lead to the suppression of protective immune responses and facilitating disease progression [89]. An increased intra-tumoral abundance of neutrophil has been shown in CRC [90], and elevated neutrophil/lymphocyte ratio (NLR) in peripheral blood of advanced CRCs is related to unfavorable prognostic aspects [91]. By frequently colocalizing with CD8^+^ T cells, neutrophils could also irritate CD8^+^ T cell response to T cell receptor priming, thus reflecting that neutrophils might have notably anti-oncogenic efficacy [92]. Thus far, the roles of neutrophils and other immune cells in CRC progression have not been fully elucidated. The investigation in-depth, presented herein, opens new avenues for understanding the relationship between immune cells and the progression of CRC.

Among the seven IRGs in the classifier, NRG1 was considered as a protective factor for CRCs while FABP4, AMH, GRP, INHBB, UCN and MC1R were risk factors for CRCs. These IRGs have been previously reported to be involved in tumorigenesis. The growth factor neuregulin 1 (NRG1) comprises of an epidermal growth factor (EGF)-like domain that binds to human tyrosine kinases of the ErbB/HER receptor family, contributing to heterodimerization and activation of the ErbB-mediated downstream signaling pathways [93]. CRC is an NRG1 fusion-positive tumor [94, 95], in which the expression of NRG1 III is significantly upregulated and negatively correlated with lymph node metastasis [96], implying a satisfactory prognosis. Primarily expressed in the adipocytes and macrophages [97], fatty acid binding protein 4 (FABP4) is involved in lipid transfer between adipocytes and tumor cells, provoking the fatty acid oxidation to induce tumor growth [98, 99]. The elevated expression of FABP4 was confirmed as a robust risk factor for the progression of CRC in a Chinese cohort [100], while an *in-silico* study also uncovered that FABP4 imposed conceivably poor prognosis on CRCs [25]. Herein, FABP4 harbored detrimental effects on CRCs and the strong interaction between the FABP4 expression and macrophages was also manifested in our study, supporting FABP4’s crosstalk with macrophages in the TIME. As a corticotropin-releasing factor-related peptide, urocortin (UCN) participated in gastrointestinal motor and visceral pain during stress response [101]. In the current study, UCN was correlated with poor CRC prognosis, in tandem with anteriorly proposed CRC signature [25, 102, 103]. The melanocortin-1 receptor (MC1R) has been regarded as an adverse parameter for survival in CRC [102]. Nevertheless, the specific implication of MC1R in CRC is rarely known. Patients carrying the MC1R variants are presented with elevated melanoma risk, and MC1R had been a therapeutic target for melanoma [104, 105]. Consequently, preclinical studies on the importance of MC1R in the development of CRC are needed. Anti-Müllerian hormone (AMH) is a member of the TGFβ family that engages in cell proliferation, differentiation, and apoptosis in normal tissues [106]. AMH was positively related to the risk of breast cancer [107], and the downregulation of AMH lower the risk of CRC was forecasted in two bioinformatic analyses [108, 109]. The inhibin subunit beta B (INHBB) is a subunit of the activin B, a functional cytokine of the TGFβ superfamily [110, 111]. INHBB is upregulated and exerts tumorigenic activity in a variety of malignant tumors ranging from oral cancer [112] to endometrial cancer [113], prostate cancer [114], and thyroid cancer [115]. In our model, elevated INHBB expression predicted an adverse outcome. Analogously, Yuan et al. indicated that the expression of INHBB was enhanced in CRC tissue, bringing about worse OS and disease-free survival (DFS) [116]. As a subtribe of the bombesin (BN)-like peptide family, gastrin-releasing peptide (GRP) is principally served as gastrointestinal hormone and neurotransmitter [117, 118]. GRP modulates the growth and differentiation of numerous human tumors including CRC [119, 120]. The GRP receptor (GRPR) has been shown to be overexpressed in human CRCs, when compared to normal colonic epithelial cells [121, 122]. Moreover, GRP and the co-expression of GRPR acted in differentiation, with the highest levels observed in well-differentiated CRC cells [123]. BN/GRP antagonists, such as RC-3095 and RC-3940-II, have been reported to exert anti-tumor activities in *in-vitro* and *in-vivo* mouse xenografts [124, 125]. RC-3940-II also exerted potent anti-neoplastic activity on the human CRC cell lines both *in vitro* and *in vivo* [126]. Li et al. pointed out that GRP could predict the prognosis of DFS in CRC [127], uncovering its involvement in the prognosis and survival of CRC. Bedke et al. demonstrated that GRP and GRPR were mainly expressed by TAMs in renal cell carcinomas (RCC) [128], accordantly, the current study indicated that the expression of GRP was positively correlated with the degree of macrophage infiltration. Briefly, these compelling evidences for the significance of GRP show great potential at unmasking the malignancy-associated roles of TAMs in CRC.

Three drugs, including AZD4547, Cytochalasin B, and S-crizotinib, harbored more notable anti-neoplastic activity in the immune risk score-high group. Intriguingly, high-risk specific agents are all anti-tumor targeted compounds, and a striking consistency was shown between the mechanism of action (MOA) of these chemical entities and enriched signatures obtained from GSEA. As prominent segment in the TME composed of cancer cells and stromal or immune cells, CAFs crosstalk with tumor cells contributes to the progression of tumor [129]. Overexpression of the fibroblast growth factor receptor-1 (FGFR-1) has been correlated with liver metastasis in CRC [130]. The fibroblast growth factor 1 (FGF1)/FGFR-3 signaling mediates migration and invasion in CRC, and activated fibroblasts upregulate the expression of FGF1 [131]. AZD4547 is an orally potent and highly selective tyrosine kinase inhibitor (TKI) targeted FGFR 1-3 [132]. Preclinical data recapitulates that AZD4547 possesses anti-oncogenic activity against various tumors, such as gastric [133], lung [134], and pancreatic [135] cancers. Yao et al. reported that AZD4547 delayed CRC tumor growth *in vitro*, and its activity was in close interaction with the expression level of FGFR [136]. In our study, the infiltration of fibroblasts was apparently higher in high-risk score CRCs, compellingly argue for clinical investigations of AZD4547 for treating high-risk specific CRCs. Cytochalasin B is a common microfilament-disrupting compound that impacts various cellular physiological processes mediated by F-actin, encompassing cell motility, endocytosis and adherence [137–139]. Treating human CRC SW480 cells with cytochalasin B attenuated the downregulation of E-cadherin expression [140]. Indeed, the loss or dysregulation of E-cadherin expression expedites the growth, invasion, and drug resistance in CRC cells [141, 142]. EMT, a morphogenetic process whereby epithelial cells transform to the mesenchymal phenotype, critically engaged in tumorigenesis and cancer progression [143]. In tumor, the expression of epithelial markers, E-cadherin particularly, is downregulated during the process of EMT, ultimately destroying cell adhesion, promoting cell motility and stages of cancer [144, 145]. Conversely, inhibited EMT as evidenced by the elevated expression of E-cadherin exerts suppressive effects on the growth and invasion of human CRC via the Wnt/β-catenin signaling [146, 147]. c-MET/RON activation initiates many facets of cellular responses covering motility, proliferation, EMT, and angiogenesis [148, 149]. Typically, c-Met and RON signaling irritate angiogenesis through the interplay with vascular endothelial growth factor (VEGF) stimulated by hypoxia-inducible factor 1-alpha (HIF-1α). Crizotinib is an extensively functioning, small-molecule TKI clinically approved for treating non-small-cell lung cancer (NSCLC) patients [150]. In a three-dimensional CRC culture system, Li et al. found that crizotinib restored cetuximab sensitivity in the HCA-7 CRC cell line [151]. By inhibiting c-MET/RON/ALK/MTH1, S-crizotinib is an optical isomer of a clinical anticancer compound, R-crizotinib, with inhibited efficacy in suppressing MTH1 compared to S-crizotinib [152, 153]. Previous evidence suggests that MTH1 inhibition via S-crizotinib induced an increase in DNA single strand breaks as well as activated DNA repair in SW480 cells [153]. In human cells, acute MTH1 inhibition enables p53-dependent cellular senescence upon hyperoxia [154]. Moreover, MTH1 is pivotal in RAS-driven oncogenesis and its overexpression accelerates the spectrum of RAS-driven carcinogenic transformation [155]. Notably, elevated expression of MTH1 enhances the transformation of immortalized cells through RAS and maintains pro-oncogenic phenotype, EMT [156, 157]. Collectively, we postulate that AZD4547, Cytochalasin B and S-crizotinib are attractive compounds for further pre-clinical investigations and could be promising novel anti-cancer agents for IRGs risk score-high CRCs.

Immunotherapy, with special regard to ICIs, has attracted great interest in oncotherapy and has been applied in clinical practice for a variety of malignancies. Pembrolizumab and nivolumab that inhibited PD-1 and ipilimumab targeted CTLA-4 have been approved by the United States Food and Drug Administration (FDA) as second-line treatment in MSI-high and dMMR advanced CRCs. Focused on the findings from KEYNOTE 028 [13] and CheckMate 142 [11], solely a modest percentage of advanced CRCs harbored a persistent and stable response during the ICI therapy, with response rate at 30-55%. Therefore, it is of great clinical significance to develop a biomarker for predicting immunotherapeutic efficacy. In this study, we confirmed that CRCs with a low-risk immune signature were markedly related with enhanced response to ICIs targeted PD-1, PD-L1 and CTLA-4, while the immune score-high CRCs exhibited nonresponse to PD-1 inhibitor and MAGE-A3 based immunotherapy. These findings illustrated that the IRGs-based risk score could be served as a practical tool for assessing immunotherapeutic efficacy in CRC, in accordance with a recent study on the immune signature score for colon cancer [24]. Compared to the immune high-risk subclass, the low-risk subclass exhibited significantly higher infiltration of anti-tumor immune cells and expression of immune checkpoint genes, which may account for diverse responses between the two subclasses. Furthermore, the GSEA of hallmark gene sets indicated that the upregulated genes in the high-risk subgroup were enriched in Wnt/β-catenin signaling, consistent with previous findings that the activation of tumor-intrinsic β-catenin pathway could induce T-cell exclusion, thereby causing resistance to PD-L1 or CTLA-4 blockade immunotherapy [158]. Thus, altered Wnt/β-catenin signaling activation may be associated with immunotherapeutic resistance in CRC.

However, there are still some limitations in this study. Firstly, we attempted to obtain abundant CRC cohorts to achieve more reliable results with sufficient sample size. But the intra-tumor or intra-patient heterogeneity of the TIME in CRCs was not fully considered, which impacted the effect of chemotherapy and immunotherapy. Secondly, the median cutoff of IRGs risk score was utilized to stratify the CRC samples into high-risk subtype and low-risk subtype, and the optimal cutoff of the risk score is needed to best classify the CRCs. Thirdly, all the conclusions in this study were inferred from *in-silico* analyses, and further *in-vitro* or *in-vivo* experiments and clinical validations are needed to promote the clinical application of our findings. Finally, due to the paucity of CRC cohorts treated with immunotherapy, more prospective clinical studies are required to further verify this novel IRGs-based signature in CRCs.

## CONCLUSIONS

The IRGs signature is valuable for its correlation with immune infiltration, and the association between the risk score and OS in the integrated analysis of CRC cohorts suggests that it is a robust prognostic biomarker for CRC. This IRGs model harbors crucial clinical practicality in both high- and low- risk CRCs who had failed first-line treatment or progressed. For immune low-risk score patients, clinicians could adopt ICIs targeted PD-1, PD-L1 and CTLA-4 as well as MAGE-A3 immunotherapy strategies to avoid excessive treatment, so these CRCs could acquire a better quality of life with a favorable prognosis. For immune high-risk score patients, AZD4547, Cytochalasin B and S-crizotinib might be used in cases of immunotherapeutic resistance. Generally, our finding provides new insights into determining the prognosis of CRCs, and sheds new light on tailoring CRCs with precise treatment.

## Supplementary Material

Supplementary Figures

Supplementary Table 1

Supplementary Tables 2, 3 and 4

Supplementary Table 5

Supplementary Table 6

Supplementary Table 7
