# Supplementary material for: Identification of a novel immune signature for optimizing prognosis and treatment prediction in colorectal cancer
Source: Aging (Albany NY). 2021 Dec 13;13(23):25518–49. doi: 10.18632/aging.203771 (PMC8714135; doi:10.18632/aging.203771)
Supplement: Supplementary Figures [file aging-13-203771-s001.pdf]

SUPPLEMENTARY FIGURES

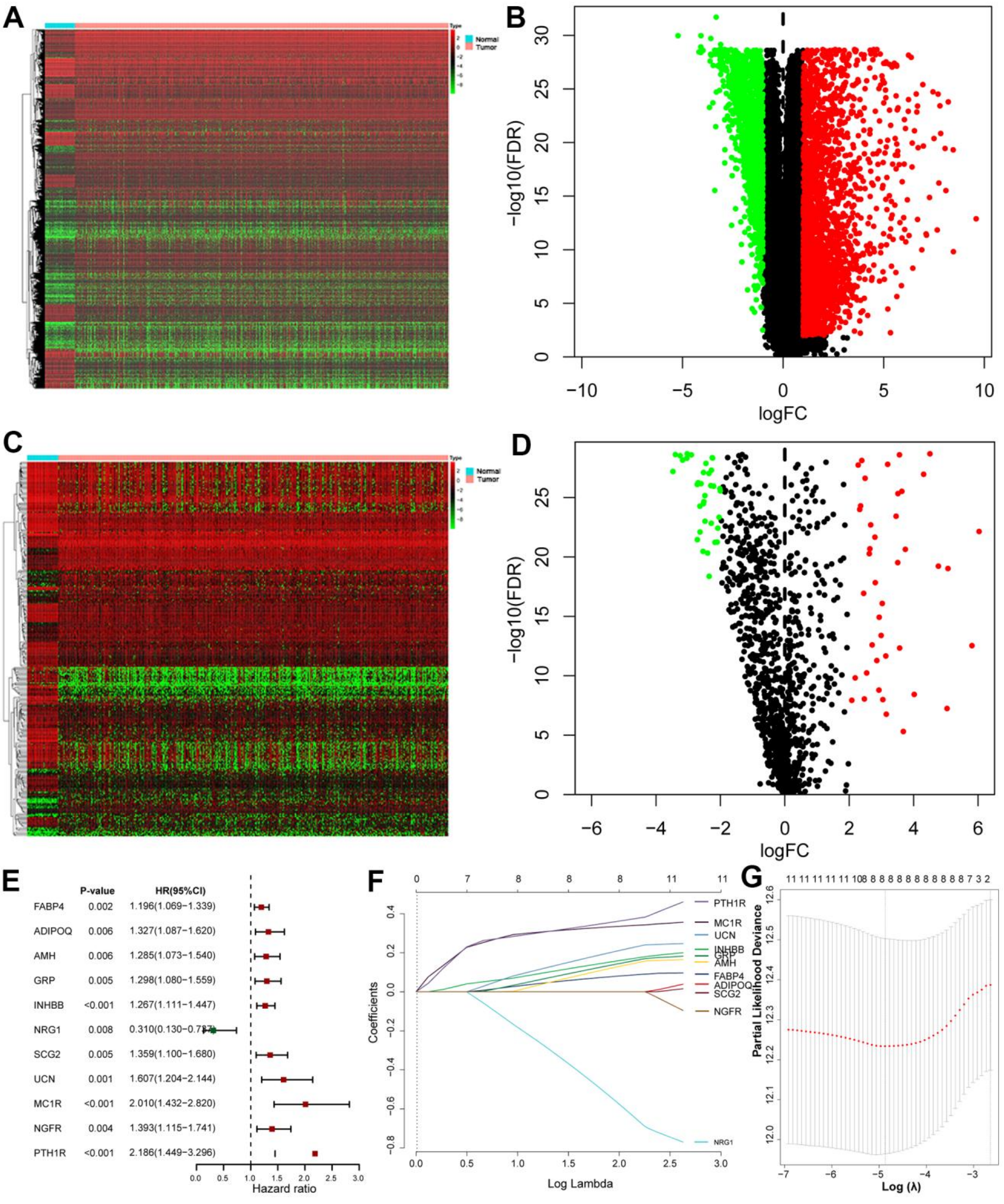

Supplementary Figure 1. Construction of the IRGs-based risk score model through TCGA training cohort. (**A**, **B**) Differentially expressed genes in CRC. (**C**, **D**) Differentially expressed IRGs in CRC. (**E**) Forest plot of PRIRGs via univariate Cox regression analysis. (**F**) lasso coefficient profiles of 11 PRIRGs. (**G**) Partial likelihood deviance of variables estimated by the lasso regression algorithm. CRC, colorectal cancer; IRGs, immune-related genes; PRIRGs, prognosis-related IRGs.

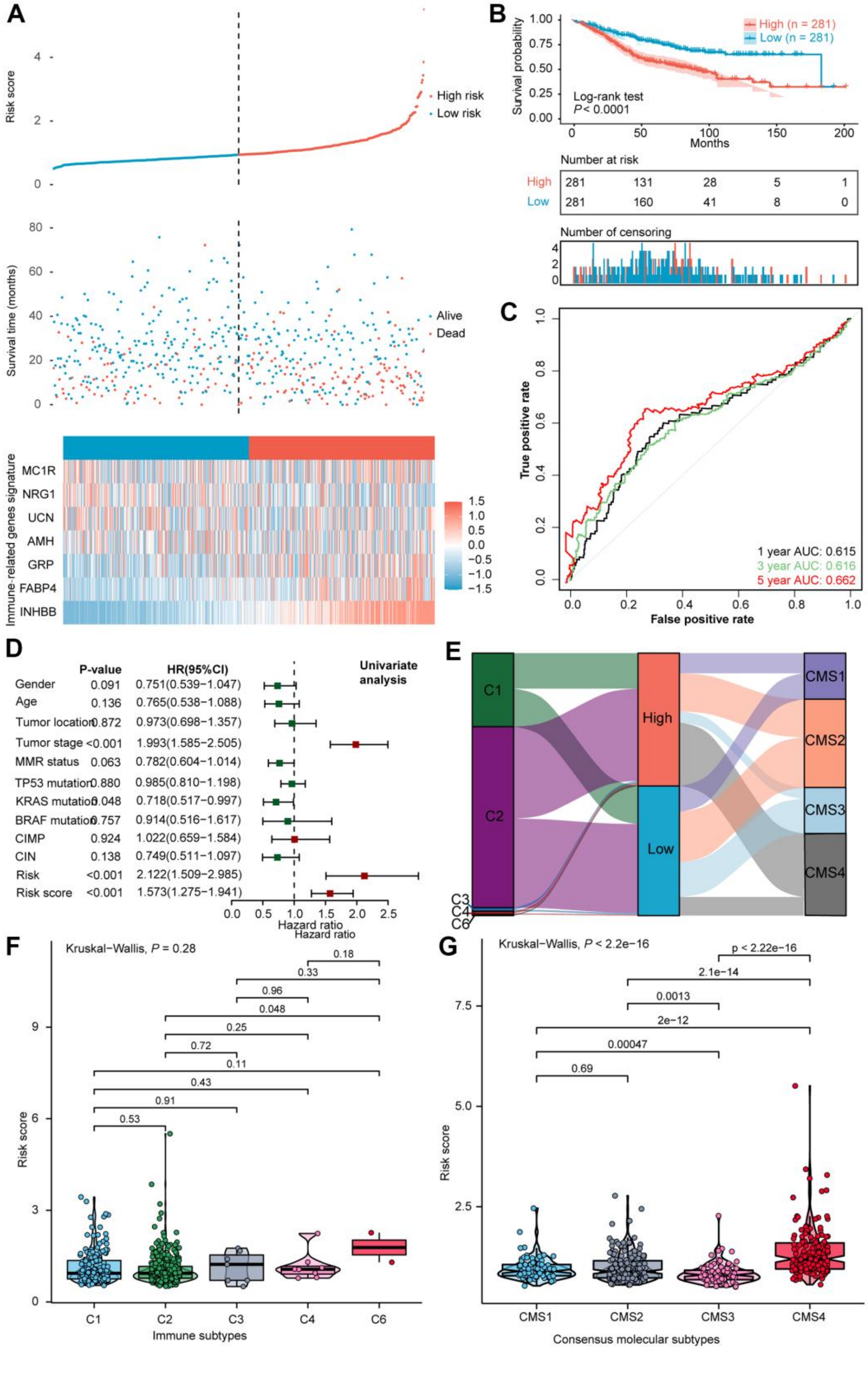

Supplementary Figure 2. Exploration of the predictive power and clinical characteristics of seven IRGs signature in the validation cohort. (**A**) Distribution of risk score, survival status, and the expression of seven IRGs of CRCs. (**B**) Kaplan-Meier survival curve of the high- and low- risk subgroups. (**C**) ROC curve analysis of IRGs in the validation cohort. (**D**) Univariate Cox analysis of prognostic factors and OS of CRCs. (**E**) Alluvial diagram for the two subtypes versus different immune subtypes and CMS. (**F**) Violin plot illustrated the correlation between risk score and immune subtypes, and (**G**) CMS. AUC, area under the curve; OS, overall survival; CRC, colorectal cancer; IRGs, immune-related genes; ROC, receiver operating characteristic; CMS, consensus molecular subtypes.

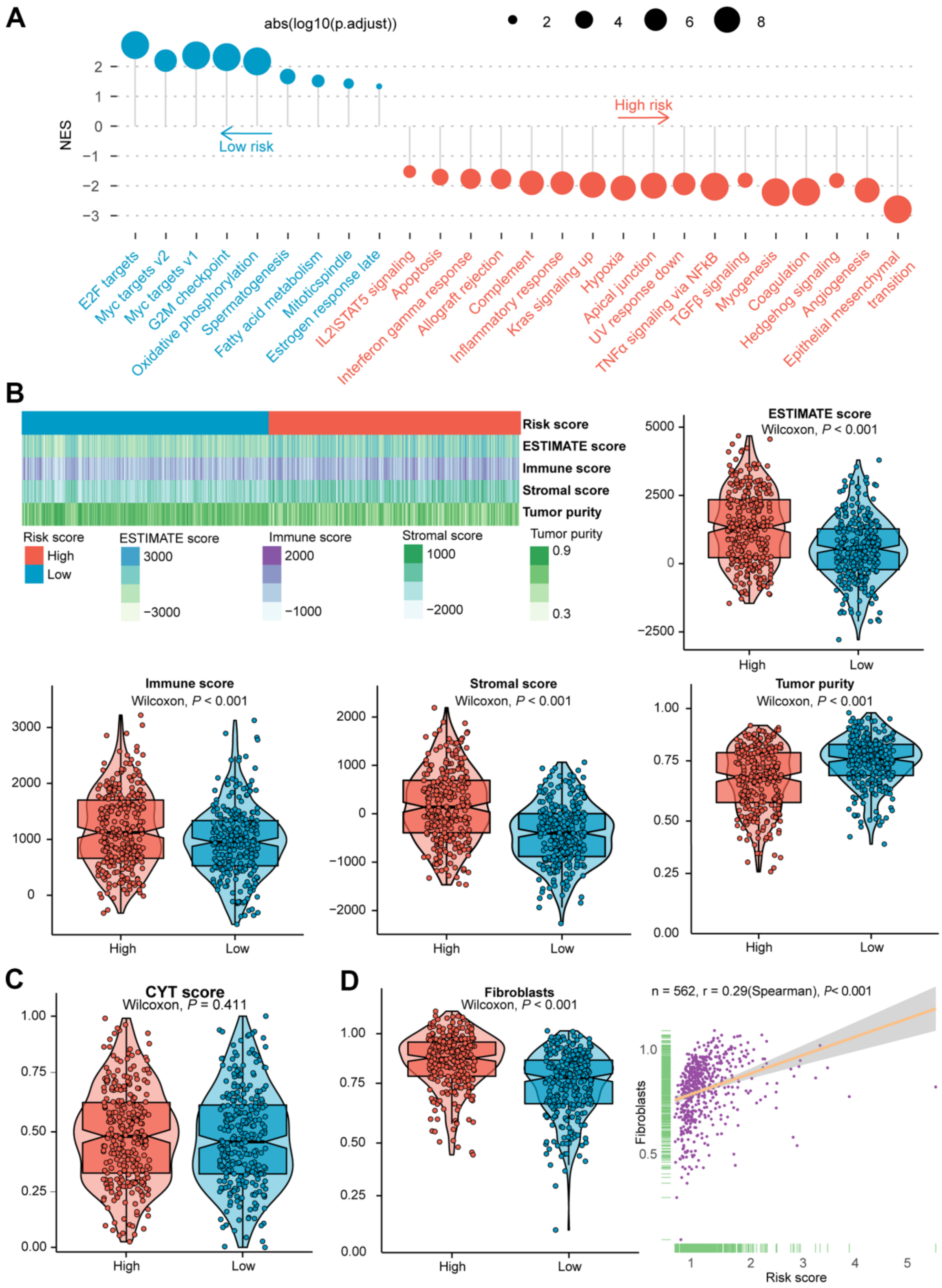

Supplementary Figure 3. Evaluation of the role of the risk score in the validation cohort. (**A**) Results of GSEA of the high-risk group (red) compared with the low-risk group (blue). Color toward gray represents no statistical significance. (**B**) Heatmap and violin plots of the ESTIMATE score, immune score, stromal score, tumor purity between high- and low- risk subtypes. (**C**) Violin plot of the CYT score between high- and low- risk subtypes. (**D**) Violin plot of fibroblasts between two subtypes, and the association between risk score and the NES of fibroblasts. Statistical significance at the level of ns ≥ 0.05, * < 0.05, ** < 0.01 and *** < 0.001. GSEA, gene set enrichment analysis; CYT, cytolytic activity; NES, normalized enrichment score.

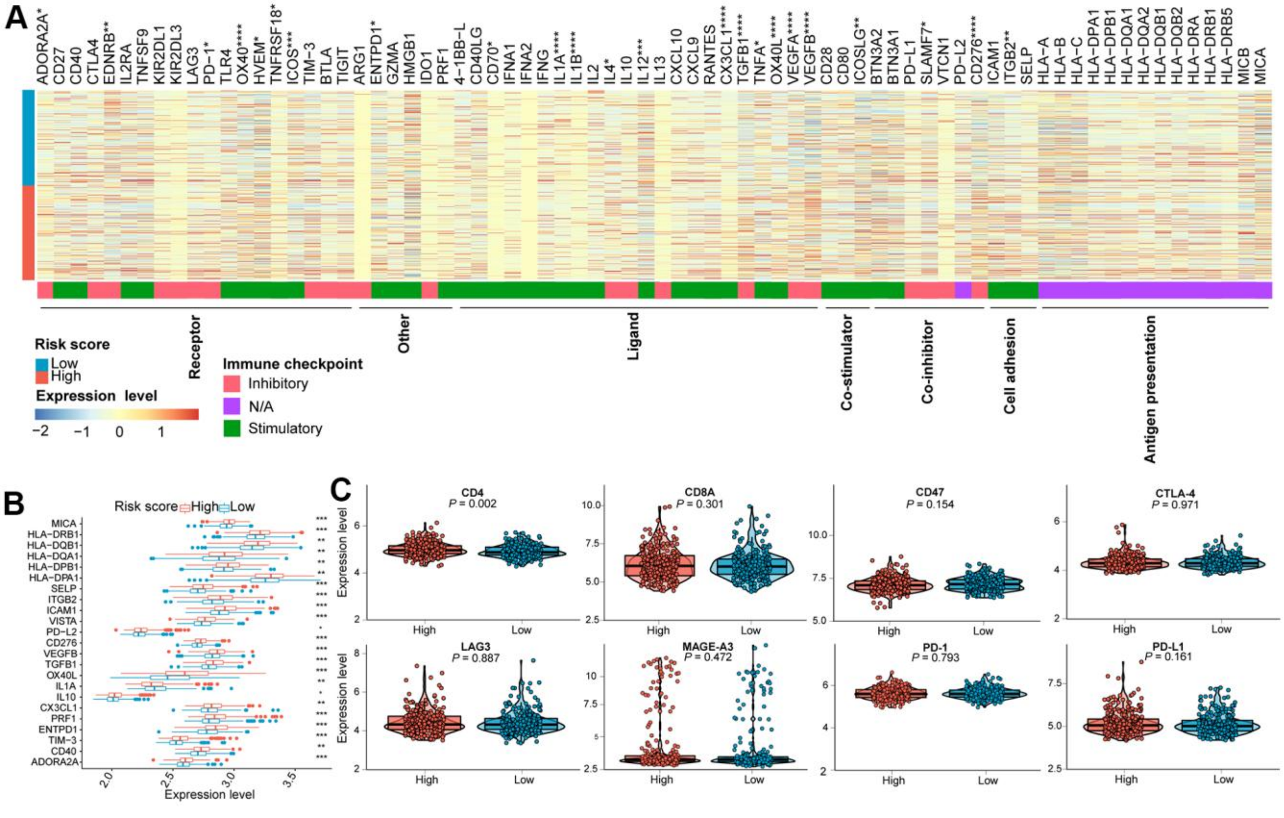

Supplementary Figure 4. (**A**) The differential expression levels of immune checkpoint molecules within distinctive subgroups in the TCGA CRCs. (**B**) The differential expression level of immune checkpoint molecules between two subclasses with statistical significance in the validation cohort. (**C**) Violin plots of the CD4, CD8A, CD47, CTLA4, LAG3, MAGE-A3, PD-1 and PD-L1 expression levels for two subtypes in the validation cohort.

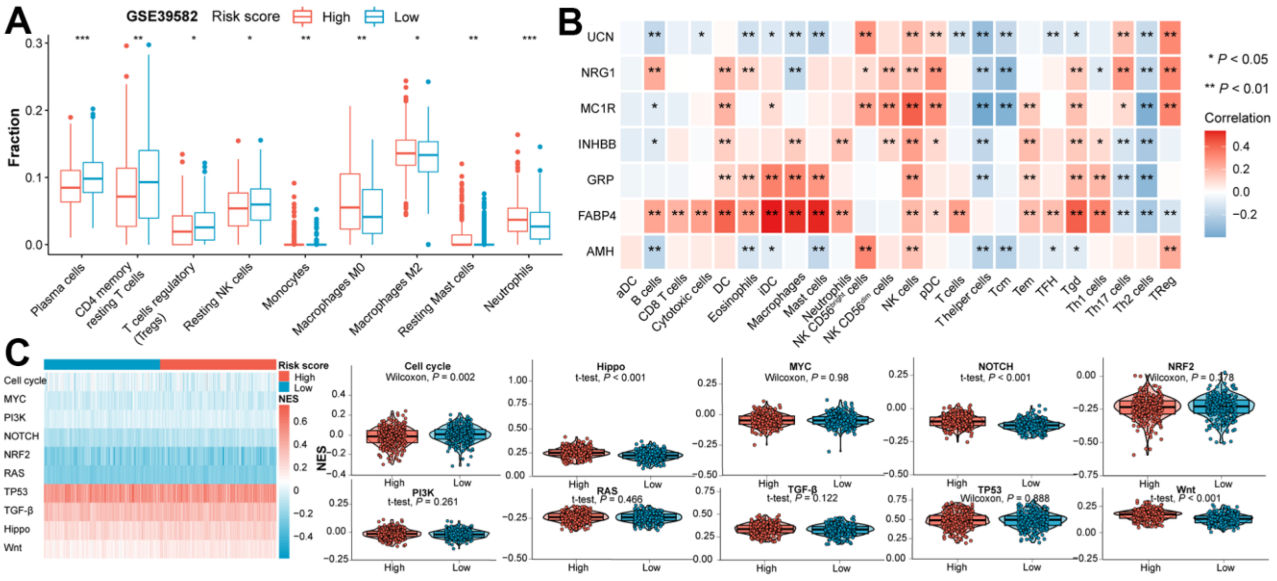

Supplementary Figure 5. (**A**) Immune infiltration between high- and low- risk subtypes with statistical significance in the validation cohort. (**B**) Correlation analysis between the expression of seven IRGs and the infiltration of immune cells in the validation cohort. (**C**) Heatmap and violin plots of the NES of 10 oncogenic pathways between two subtypes in the validation cohort. Statistical significance at the level of ns ≥ 0.05, * < 0.05, ** < 0.01 and *** < 0.001.
