# Supplementary material for: Identification of a novel immune signature for optimizing prognosis and treatment prediction in colorectal cancer
Source: Aging (Albany NY). 2021 Dec 13;13(23):25518–49. doi: 10.18632/aging.203771 (PMC8714135; doi:10.18632/aging.203771)
Supplement: Supplementary Tables 2, 3 and 4 [file aging-13-203771-s003.pdf]

SUPPLEMENTARY TABLES

Supplementary Table 2. Eight PRIRGs filtered out by lasso regression.

IRGs	Coefficient
FABP4	0.0813685612698171
AMH	0.131513420971701
GRP	0.146281413219288
INHBB	0.160968056160568
NRG1	-0.582958093399237
UCN	0.213129186143968
MC1R	0.335915826080654
PTH1R	0.363374360149091

Supplementary Table 3. Overall information of seven IRGs in the signature.

Ensembl ID	IRGs	Coefficient	HR (95% CI)	*P*-value
ENSG00000170323	FABP4	0.139	1.15 (1.021-1.294)	0.021
ENSG00000104899	AMH	0.176	1.193 (0.962-1.479)	0.109
ENSG00000134443	GRP	0.207	1.229 (1.014-1.491)	0.036
ENSG00000163083	INHBB	0.211	1.235 (1.064-1.433)	0.006
ENSG00000157168	NRG1	-0.691	0.501 (0.216-1.164)	0.108
ENSG00000163794	UCN	0.274	1.315 (0.929-1.863)	0.122
ENSG00000258839	MC1R	0.366	1.442 (0.952-2.183)	0.084

Supplementary Table 4. Clinical characteristics of the TCGA cohort and the GSE39582 cohort.

	TCGA cohort		
**Variable**	**Low (n = 208)**	**High (n = 229)**	**Overall (n = 437)**
**Gender**			
Male	113 (54.3%)	119 (52.0%)	232 (53.1%)
Female	95 (45.7%)	110 (48.0%)	205 (46.9%)
**Race**			
White	107 (51.4%)	124 (54.1%)	231 (52.9%)
Black or african american	25 (12.0%)	30 (13.1%)	55 (12.6%)
Others	76 (36.5%)	75 (32.8%)	151 (34.6%)
**Age**			
>= 65	107 (51.4%)	139 (60.7%)	246 (56.3%)
<65	101 (48.6%)	90 (39.3%)	191 (43.7%)
**History of colon polyps**			
No	133 (63.9%)	134 (58.5%)	267 (61.1%)
Yes	48 (23.1%)	54 (23.6%)	102 (23.3%)
NA	27 (13.0%)	41 (17.9%)	68 (15.6%)
**Tumor site**			
Left	130 (62.5%)	117 (51.1%)	247 (56.5%)
Right	78 (37.5%)	112 (48.9%)	190 (43.5%)
**Tumor stage**			
I	48 (23.1%)	29 (12.7%)	77 (17.6%)
II	79 (38.0%)	79 (34.5%)	158 (36.2%)
III	59 (28.4%)	73 (31.9%)	132 (30.2%)
IV	22 (10.6%)	48 (21.0%)	70 (16.0%)
**MSI status**			
MSI-H	33 (15.9%)	24 (10.5%)	57 (13.0%)
MSI-L	31 (14.9%)	42 (18.3%)	73 (16.7%)
MSS	144 (69.2%)	163 (71.2%)	307 (70.3%)
**MMR status**			
pMMR	133 (63.9%)	140 (61.1%)	273 (62.5%)
dMMR	25 (12.0%)	29 (12.7%)	54 (12.4%)
NA	50 (24.0%)	60 (26.2%)	110 (25.2%)
**TP53 mutation**			
Mutant	126 (60.6%)	143 (62.4%)	269 (61.6%)
Wildtype	82 (39.4%)	86 (37.6%)	168 (38.4%)
**KRAS mutation**			
Mutant	82 (39.4%)	110 (48.0%)	192 (43.9%)
Wildtype	126 (60.6%)	119 (52.0%)	245 (56.1%)
**BRAF mutation**			
Mutant	24 (11.5%)	25 (10.9%)	49 (11.2%)
Wildtype	184 (88.5%)	204 (89.1%)	388 (88.8%)
**EGFR mutation**			
Mutant	1 (0.5%)	8 (3.5%)	9 (2.1%)
Wildtype	207 (99.5%)	221 (96.5%)	428 (97.9%)
**NRAS mutation**			
Mutant	8 (3.8%)	18 (7.9%)	26 (5.9%)
Wildtype	200 (96.2%)	211 (92.1%)	411 (94.1%)
**APC mutation**			
Mutant	169 (81.2%)	177 (77.3%)	346 (79.2%)
Wildtype	39 (18.8%)	52 (22.7%)	91 (20.8%)

	GSE39582 cohort		
**Variable**	**Low (n = 220)**	**High (n = 222)**	**Overall (n = 442)**
**Gender**			
Male	122 (55.5%)	117 (52.7%)	239 (54.1%)
Female	98 (44.5%)	105 (47.3%)	203 (45.9%)
**Age**			
>= 65	135 (61.4%)	147 (66.2%)	282 (63.8%)
<65	85 (38.6%)	75 (33.8%)	160 (36.2%)
**Tumor location**			
Proximal	83 (37.7%)	96 (43.2%)	179 (40.5%)
Distal	137 (62.3%)	126 (56.8%)	263 (59.5%)
**Tumor stage**			
I	14 (6.4%)	14 (6.3%)	28 (6.3%)
II	113 (51.4%)	104 (46.8%)	217 (49.1%)
III	73 (33.2%)	76 (34.2%)	149 (33.7%)
IV	20 (9.1%)	28 (12.6%)	48 (10.9%)
**MMR status**			
pMMR	168 (76.4%)	168 (75.7%)	336 (76.0%)
dMMR	34 (15.5%)	27 (12.2%)	61 (13.8%)
NA	18 (8.2%)	27 (12.2%)	45 (10.2%)
**TP53 mutation**			
Mutant	68 (30.9%)	70 (31.5%)	138 (31.2%)
Wildtype	69 (31.4%)	55 (24.8%)	124 (28.1%)
NA	83 (37.7%)	97 (43.7%)	180 (40.7%)
**KRAS mutation**			
Mutant	86 (39.1%)	90 (40.5%)	176 (39.8%)
Wildtype	134 (60.9%)	132 (59.5%)	266 (60.2%)
**BRAF mutation**			
Mutant	19 (8.6%)	23 (10.4%)	42 (9.5%)
Wildtype	201 (91.4%)	199 (89.6%)	400 (90.5%)
**CIMP**			
Negative	176 (80.0%)	188 (84.7%)	364 (82.4%)
Positive	44 (20.0%)	34 (15.3%)	78 (17.6%)
**CIN**			
Negative	57 (25.9%)	45 (20.3%)	102 (23.1%)
Positive	163 (74.1%)	177 (79.7%)	340 (76.9%)
