# Supplementary material for: Identification of a novel immune signature for optimizing prognosis and treatment prediction in colorectal cancer
Source: Aging (Albany NY). 2021 Dec 13;13(23):25518–49. doi: 10.18632/aging.203771 (PMC8714135; doi:10.18632/aging.203771)
Supplement: Supplementary Table 7 [file aging-13-203771-s006.pdf]

Supplementary Table 7. List of potential therapeutic agents for CRC patients with IRGs signature high-risk score.

Name	Source	MOA	Target	Evidence for CRC treatment
AT7867	CTRP	Akt inhibitors	Akt	PMID: 28081222
AZD4547	CTRP	FGFR inhibitors	FGFR	PMID: 25691251
BRD-K16147474	CTRP	NA	NA	NA
cytochalasin B	CTRP	excitatory proteins inhibitors	cytoskeleton/endocytosis	PMID: 16287074
PLX-4032	CTRP	B-raf^{V600E} inhibitors	B-raf	PMID: 29326440
SGX-523	CTRP	Met kinase inhibitors	c-Met	NA
PLX-4720	CTRP	B-raf^{V600E} inhibitors	B-raf	PMID: 25381152/26351322
TG-101348	CTRP	JAK2 inhibitors	JAK2/STAT3/PIM1 pathway	PMID: 32346607
lovastatin	CTRP	HMG-CoA reductase inhibitors	HMG-CoA reductase	PMID: 24945998
BRD-K37390332	CTRP	NA	NA	NA
AMG458	PRISM	MET/RON inhibitors	MET/RON	NA
LE135	PRISM	RARβ antagonist	RARβ	NA
mevastatin	PRISM	HMG-CoA reductase inhibitors	HMG-CoA reductase	PMID: 11408350
creatine	PRISM	NA	NA	NA
S-crizotinib	PRISM	ALK/RON/c-MET, MTH1 inhibitors	ALK/RON/c-MET, MTH1	PMID: 24695225/28320945
colforsin daproate	PRISM	adenylate cyclase agonist	adenylate cyclase	NA
erythritol	PRISM	NA	cytidylyltransferase	NA
CHIR-98014	PRISM	GSK3 inhibitors	GSK-3α and GSK-3β	NA
epinephrine	PRISM	adrenergic receptor agonist	adrenergic receptor	NA
tandutinib	PRISM	FLT3 inhibitors	Akt/mTOR pathway	PMID: 23427297

MOA, mechanism of action.
